# Biallelic variants in coenzyme Q10 biosynthesis pathway genes cause a retinitis pigmentosa phenotype

**DOI:** 10.1038/s41525-022-00330-z

**Published:** 2022-10-20

**Authors:** Neringa Jurkute, Francesca Cancellieri, Lisa Pohl, Catherina H. Z. Li, Robert A. Heaton, Janine Reurink, James Bellingham, Mathieu Quinodoz, Georgia Yioti, Maria Stefaniotou, Marianna Weener, Theresia Zuleger, Tobias B. Haack, Katarina Stingl, Genomics England Research Consortium, J. C. Ambrose, P. Arumugam, R. Bevers, M. Bleda, F. Boardman-Pretty, C. R. Boustred, H. Brittain, M. A. Brown, M. J. Caulfield, G. C. Chan, A. Giess, J. N. Griffin, A. Hamblin, S. Henderson, T. J. P. Hubbard, R. Jackson, L. J. Jones, D. Kasperaviciute, M. Kayikci, A. Kousathanas, L. Lahnstein, A. Lakey, S. E. A. Leigh, I. U. S. Leong, F. J. Lopez, F. Maleady-Crowe, M. McEntagart, F. Minneci, J. Mitchell, L. Moutsianas, M. Mueller, N. Murugaesu, A. C. Need, P. O‘Donovan, C. A. Odhams, C. Patch, D. Perez-Gil, M. B. Pereira, J. Pullinger, T. Rahim, A. Rendon, T. Rogers, K. Savage, K. Sawant, R. H. Scott, A. Siddiq, A. Sieghart, S. C. Smith, A. Sosinsky, A. Stuckey, M. Tanguy, A. L. Taylor Tavares, E. R. A. Thomas, S. R. Thompson, A. Tucci, M. J. Welland, E. Williams, K. Witkowska, S. M. Wood, M. Zarowiecki, Carel B. Hoyng, Omar A. Mahroo, Iain Hargreaves, F. Lucy Raymond, Michel Michaelides, Carlo Rivolta, Susanne Kohl, Susanne Roosing, Andrew R. Webster, Gavin Arno

**Affiliations:** 1 Moorfields Eye Hospital NHS Foundation Trust, London, UK; 2 Institute of Ophthalmology, University College London, London, UK; 3 Institute of Molecular and Clinical Ophthalmology Basel (IOB), Basel, Switzerland; 4 Department of Ophthalmology, University of Basel, Basel, Switzerland; 5 University Eye Hospital, Centre for Ophthalmology, University Hospital Tübingen, Tübingen, Germany; 6 Donders Institute for Brain, Cognition and Behaviour, Radboud University Medical Center, Nijmegen, the Netherlands; 7 Department of Ophthalmology, Radboud University Medical Center, Nijmegen, the Netherlands; 8 School of Pharmacy and Biomolecular Sciences, Liverpool John Moores, Liverpool, UK; 9 Department of Human Genetics, Radboud University Medical Center, Nijmegen, The Netherlands; 10 Department of Genetics and Genome Biology, University of Leicester, Leicester, UK; 11 University of Ioannina Medical School, Ioannina, Greece; 12 Oftalmic Clinical Research Organization, Moscow, Russia; 13 Institute of Medical Genetics and Applied Genomics, University of Tübingen, Tübingen, Germany; 14 Centre for Rare Diseases, University of Tübingen, Tübingen, Germany; 15 NIHR BioResource—Rare Diseases, Cambridge University Hospitals NHS Foundation Trust, Cambridge Biomedical Campus, Cambridge, UK; 16 Department of Medical Genetics, Cambridge Institute for Medical Research, University of Cambridge, Cambridge, UK; 17 Institute for Ophthalmic Research, Centre for Ophthalmology, University of Tübingen, Tübingen, Germany; 18 North Thames Genomic Laboratory Hub, Great Ormond Street Hospital for Children, London, UK; 19 Genomics England, London, UK; 20 William Harvey Research Institute, Queen Mary University of London, London, EC1M 6BQ UK

**Keywords:** Disease genetics, Hereditary eye disease

## Abstract

The aim of this study was to investigate coenzyme Q10 (CoQ_10_) biosynthesis pathway defects in inherited retinal dystrophy. Individuals affected by inherited retinal dystrophy (IRD) underwent exome or genome sequencing for molecular diagnosis of their condition. Following negative IRD gene panel analysis, patients carrying biallelic variants in CoQ_10_ biosynthesis pathway genes were identified. Clinical data were collected from the medical records. Haplotypes harbouring the same missense variant were characterised from family genome sequencing (GS) data and direct Sanger sequencing. Candidate splice variants were characterised using Oxford Nanopore Technologies single molecule sequencing. The CoQ_10_ status of the human plasma was determined in some of the study patients. 13 individuals from 12 unrelated families harboured candidate pathogenic genotypes in the genes: *PDSS1, COQ2, COQ4* and *COQ5*. The *PDSS1* variant c.589 A > G was identified in three affected individuals from three unrelated families on a possible ancestral haplotype. Three variants (*PDSS1* c.468-25 A > G, *PDSS1* c.722-2 A > G, *COQ5* c.682-7 T > G) were shown to lead to cryptic splicing. 6 affected individuals were diagnosed with non-syndromic retinitis pigmentosa and 7 had additional clinical findings. This study provides evidence of CoQ_10_ biosynthesis pathway gene defects leading to non-syndromic retinitis pigmentosa in some cases. Intronic variants outside of the canonical splice-sites represent an important cause of disease. RT-PCR nanopore sequencing is effective in characterising these splice defects.

## Introduction

Coenzyme Q10 (CoQ_10_), otherwise known as ubiquinone is a fat-soluble molecule synthesized in the inner mitochondrial membrane across all systems with higher expression in high energy demand tissues, such as liver, heart, kidney, muscle and the eye. CoQ_10_ is an essential electron carrier along the mitochondrial respiratory chain from complex I and complex II to complex III during the synthesis of ATP. Thus, deficiency of CoQ_10_ leads to dysfunction of the respiratory chain. Primary coenzyme Q10 deficiency is caused by biallelic pathogenic variants in nuclear genes important for CoQ_10_ biosynthesis. To date, a definitive relationship between the disease with a variable clinical manifestation and pathogenic variants in *PDSS1* (MIM #607429), *PDSS2* (MIM #610564), *COQ2* (MIM #609825), *COQ4* (MIM #612898), *COQ6* (MIM #614647), *COQ8A* (MIM #606980), *COQ8B* (MIM #615567), *COQ9* (MIM #612837) has been reported^1–4^. Clinical phenotyping of primary coenzyme Q10 deficiency has suggested retinopathy may be part of the syndromic phenotype in a number of cases^5–10^. A recent study reported nephropathy and retinopathy without neurological involvement in affected members of one family harbouring biallelic *COQ2* variants^11^.

Current genetic testing strategies for inherited retinal dystrophy (IRD) fail to identify pathogenic variants in a significant proportion of cases suggesting that previously unidentified genes harbouring pathogenic variants remain to be identified^12,13^. However, genome sequencing (GS) and unbiased interrogation of genomic variants offers an unparalleled opportunity to detect variants in both uncharacterised genes and non-coding regions of the genome leading to previously unknown gene/disease associations (for example the association of variants in *HGSNAT* with a non-syndromic retinal degeneration)^14,15^. Here, we report findings from genome- and exome-wide analysis of previously unsolved IRD patients and report 13 individuals from 12 families with biallelic candidate variants in CoQ_10_ biosynthesis pathway genes suggesting that defects in this pathway represent a previously unreported mechanism of non-syndromic retinopathy.

## Results

**Fig. 1:**
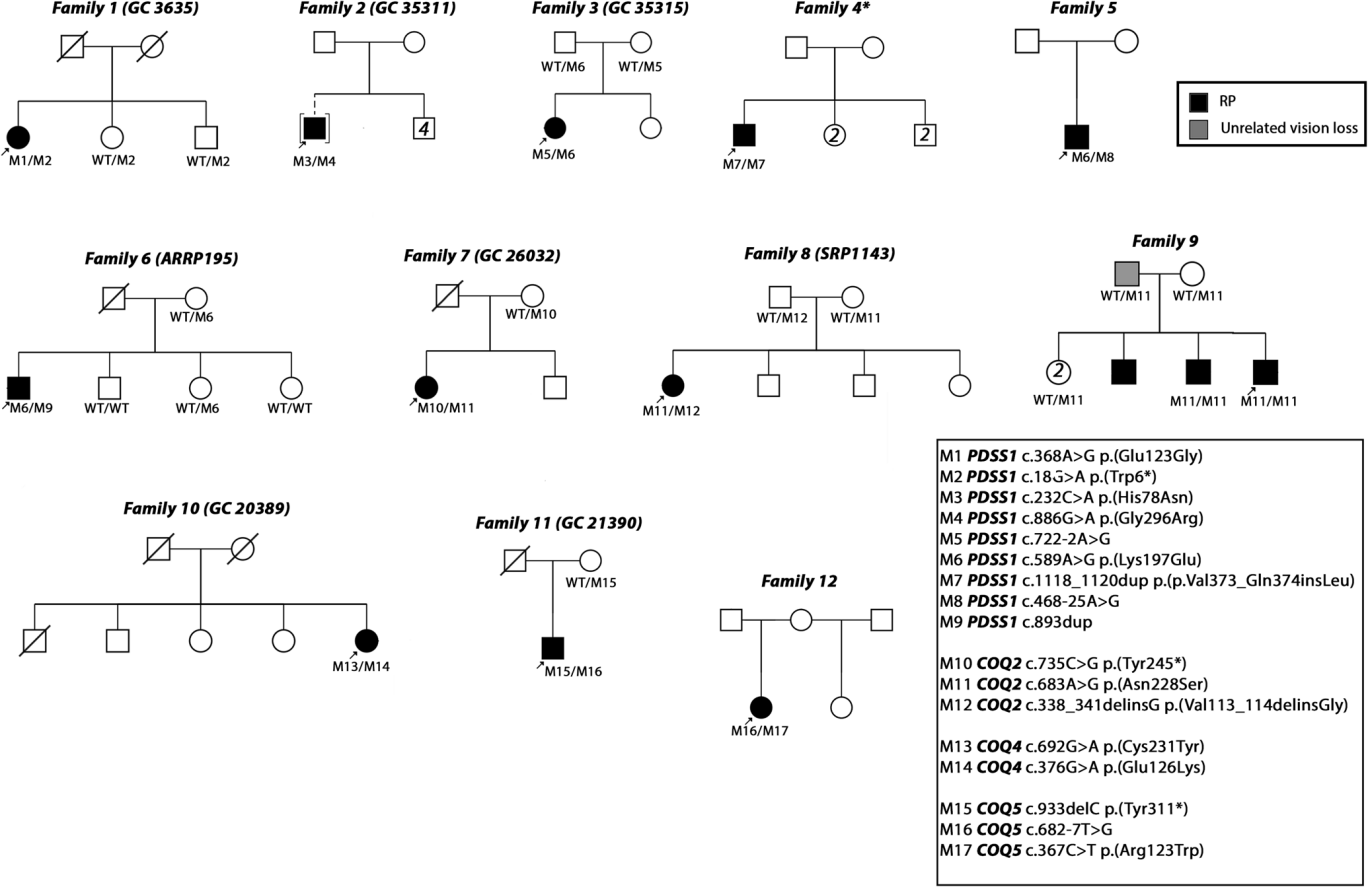
Pedigrees of families and identified candidate pathogenic variants in CoQ10 biosynthesis pathway genes. An arrow indicates the proband. Shaded symbols represent affected individuals. Different shading in family 9 indicates unrelated vision loss (not retinopathy). M – mutant, WT – wildtype.

**Fig. 2:**
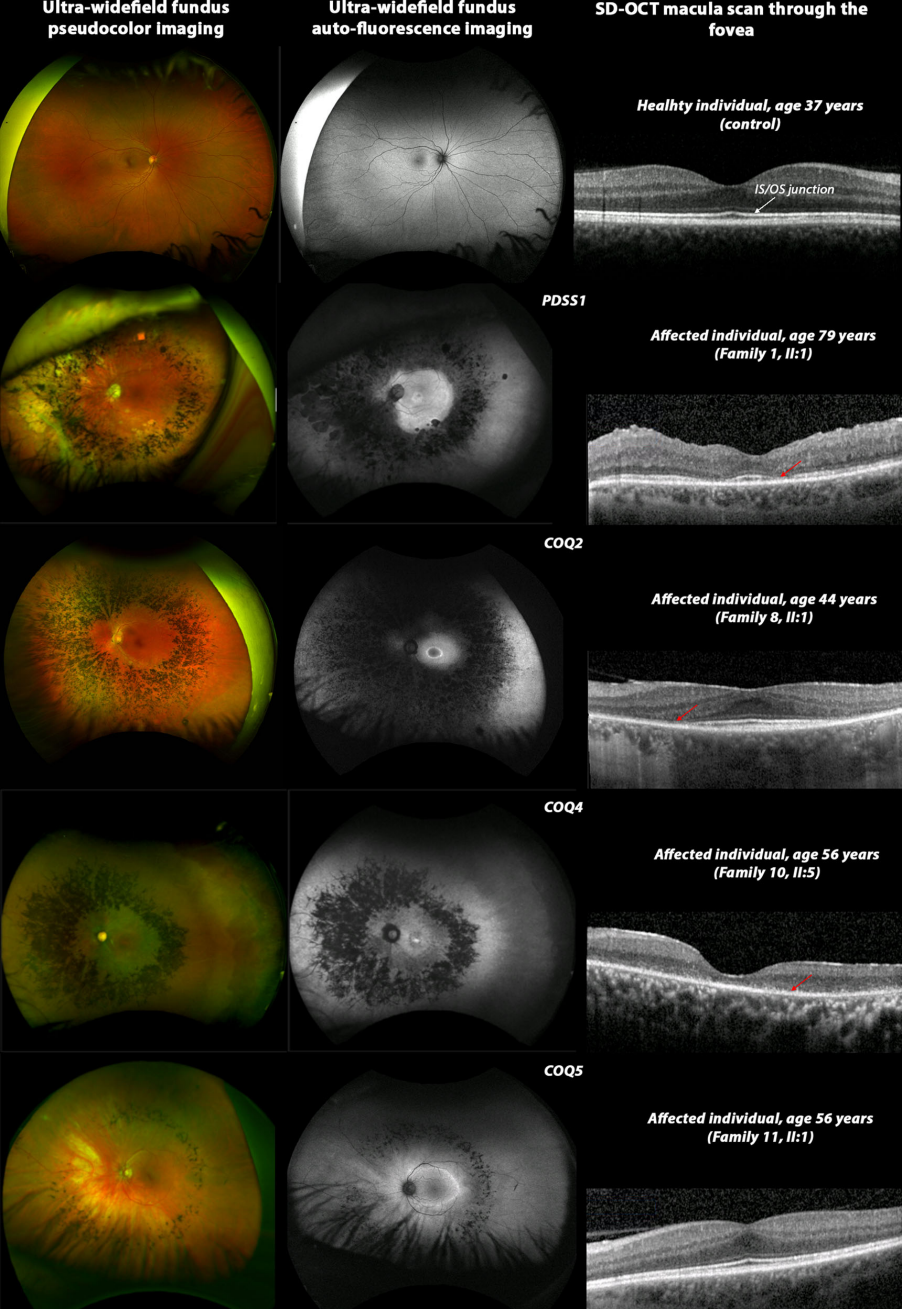
Multimodal imaging composite of control (right eye only) and four affected individuals (left eye only) carrying candidate pathogenic variants in *PDSS1*, *COQ2*, *COQ4* and *COQ5*. Ultra-widefield fundus pseudocolor imaging shows characteristic features of retinitis pigmentosa: retinal vessels attenuation, pigmentary (bone spicule) changes in mid-periphery. Ultra-widefield fundus auto-fluorescence image demonstrates hypoautofluorescence corresponding to mid-peripheral bone spicules with a hyperautofluorescence ring at the macula delineating the border between normal (inside the ring) and abnormal retina (outside the ring). SD-OCT macula scan through the fovea shows absence of inner segment/outer segment (IS/OS) junction (red arrow) in the parafoveal region in affected individuals carrying candidate pathogenic variants in *PDSS1*, *COQ2* and *COQ5*. Absence of IS/OS junction in the fovea and parafoveal region in affected individual from family 10, II:5.

Study individuals underwent virtual gene panel analysis for candidate variants in genes previously shown to be associated with posterior segment disorders, no variants were identified. Subsequent genome-wide analysis identified candidate biallelic variants in CoQ_10_ biosynthesis pathway genes in 13 affected individuals with a clinical diagnosis of retinitis pigmentosa (RP) from 12 unrelated families (Fig. 1). Biallelic variants were identified in *PDSS1* (6 families, 6 affected individuals), *COQ2* (3 families, 4 affected individuals), *COQ4* (1 family, 1 affected individual) and *COQ5* (2 families, 2 affected individuals) genes (Fig. 1, Table 1) in patients with simplex or syndromic RP (Table 2). All affected individuals had vision problems in the dark or low light conditions, constricted visual fields and relatively preserved central vision. Some of the affected individuals were diagnosed with RP later in life following referral at a routine vision check-up. Fundus examination showed retinal vessel attenuation and bone spicule pigmentation in the mid-periphery (Fig. 2). Fundus autofluorescence showed corresponding retinal pigment epithelium (RPE) atrophy in the mid-periphery with a ring of hyperautofluorescence at the macula delineating the border between functional (inside the ring) and non-functional retina (outside the ring). OCT imaging showed loss of photoreceptor outer segments in the outer macula, corresponding to the hyperautofluorescent ring, loss of RPE, as well as decreased choroidal thickness, cystoid macular oedema and epiretinal membrane in some of the affected individuals (Fig. 2 and Supplementary Fig. 1). Various degrees of hearing impairment (4/12, 33.33%) and cardiac problems (4/12, 33.33%) were the most common extraocular features observed in the study cohort. Two affected individuals carrying biallelic pathogenic variants in *COQ2* were diagnosed with renal failure, which required renal transplantation. One of them, II:1 from family-7 presented with childhood onset renal failure, which required renal transplantation at the age of 7 years and re-transplantation at the age of 30 years. For affected individual II:5 from family-9 renal transplantation was performed at the age of 55 years. A summary of clinical manifestations identified in the study cohort is presented in Table 2.

**Table 1:** Genotypes identified in study cohort.

Family ID	Gene	HGVs	HGVp	gnomAD v2 (MAF)	In silico (Mutation taster)	ACMG/AMP*	ClinVar	References
Family-1	*PDSS1*	c.368 A > G	p.(Glu123Gly)	-	Disease causing	VUS (PM2, PM3, BP1)	–	This study
(GC3635)		c.18 G > A	p.(Trp6*)	-	Disease causing	P (PVS1, PM2, PP3)	–	This study
Family-2	*PDSS1*	c.232 C > A	p.(His78Asn)	-	Disease causing	VUS (PM2, BP4)	–	This study
(GC25311)		c.886 G > A	p.(Gly296Arg)	0.00009239	Disease causing	VUS (PM2, PP3, BP1)	–	This study
Family-3	*PDSS1*	c.722-2 A > G	p.(Ala204_Ala277del); p.(Gly241Alafs*6)	0.000008799	See suppl. Table 1	P (PVS1, PM2, PP3)	–	This study
(GC25315)		**c.589** **A** > **G**	p.(Lys197Glu)	0.003499	Polymorphism	VUS (PS4, PM2, PM3, BP1, BP4)	CI	This study
Family-4	*PDSS1*	c.1118_1120dup	p.(Val373_Gln374insLeu)	-	Polymorphism	VUS (PM2, PM4, PP3)	–	This study
		c.1118_1120dup	p.(Val373_Gln374insLeu)	-	Polymorphism	VUS (PM2, PM4, PP3)		This study
Family-5	*PDSS1*	c.468-25 A > G	p.?	0.00002641	See suppl. Table 1	VUS (PM2)	–	This study
		**c.589** **A** > **G**	p.(Lys197Glu)	0.003499	Polymorphism	VUS (PS4, PM2, BP1, BP4)	CI	This study
Family-6	*PDSS1*	c.893dup	p.(Asn298Lysfs*11)	-	Disease causing	P (PVS1, PM2, PP3)	–	This study
(ARRP195)		**c.589** **A** > **G**	p.(Lys197Glu)	0.003499	Polymorphism	VUS (PS4, PM2, PM3, BP1, BP4)	CI	This study
Family -7	*COQ2*	c.735 C > G	p.(Tyr245*)	-	Disease causing	P (PVS1, PM2, PP3, PP5)	LP	This study
(GC26032)		**c.683** **A** > **G**	p.(Asn228Ser)	0.0002813	Disease causing	P (PS4, PM2, PM3, PP2, PP3)	VUS	Previously reported:^39–41^
Family-8	*COQ2*	**c.683** **A** > **G**	p.(Asn228Ser)	0.0002813	Disease causing	LP (PS4, PM2, PP2, PP3)	VUS	Previously reported:^39–41^
(SRP1143)		c.338_341delinsG	p.(Val113_114delinsGly)	-	Polymorphism	VUS (PM2, PM4, PP3)	–	This study
Family-9	*COQ2*	**c.683** **A** > **G**	p.(Asn228Ser)	0.0002813	Disease causing	LP (PS4, PM2, PP2, PP3)	VUS	Previously reported:^39–41^
		**c.683** **A** > **G**	p.(Asn228Ser)	0.0002813	Disease causing	LP (PS4, PM2, PP2, PP3)	VUS	Previously reported:^39–41^
Family-10	*COQ4*	c.692 G > A	p.(Cys231Tyr)	0.0001148	Disease causing	VUS (PM2, PP2, PP3)	–	This study
(GC20389)		c.376 G > A	p.(Glu126Lys)	0.0003508	Disease causing	VUS (PM2, PP2)	VUS	This study
Family-11	*COQ5*	c.933delC	p.(Tyr311*)	0.00001760	Disease causing	P (PVS1, PM2, PP3)	–	This study
(GC21390)		**c.682-7** **T** > **G**	p.(Gln230*); p.(Leu193Phefs*27)	0.001434	See suppl. Table 1	VUS (PM2, PM3, BP4)	–	This study
Family-12	*COQ5*	c.367 C > T	p.(Arg123Trp)	0.00003266	Disease causing	VUS (PM2, PP3)	–	This study
		**c.682-7** **T** > **G**	p.(Gln230*); p.(Leu193Phefs*27)	0.001434	See suppl. Table 1	VUS (PM2, PM3, BP4)	–	This study

Variants in bold are recurrent within the study cohort.

*CI* conflicting interpretation, *LP* likely pathogenic, *MAF* population maximum allele frequency, *P* pathogenic, *VUS* variant of uncertain significance.

*Accessed in August 2021.

**Table 2:** Clinical manifestations of study patients.

Family	No of affected	Gene	Age of onset (years)	Initial presentation, visual symptoms (age of onset)	Age of examination (years)	BCVA	Ophthalmological features^a^	Extraocular features
Family-1 (GC3635)	1	*PDSS1*	8	Hearing impairment, followed by vision problems (48). Nyctalopia documented at the age of 60 years.	79	6/6 6/9	RP, myopia, history of LE CMO, ERM peeling (55)	Hearing impairment (8), minor pericardial effusion (asymptomatic)
Family-2 (GC25311)	1	*PDSS1*	10	Decreased night vision, followed by peripheral visual field loss	30	6/18 6/18	RP, myopic astigmatism, LE CMO	Hearing impairment
Family-3 (GC25315)	1	*PDSS1*	30	Incidental finding during routine check-up (30), asymptomatic.	55	6/12 1/60	RP, LE lamellar hole (43), early bilateral cataracts (52)	Hearing impairment (mild, formally not investigated)
Family-4^b^	1	*PDSS1*	2	Hearing impairment, followed by vision problems (15).	NA	ND	RD	Hearing impairment (2), unspecified chest pain (11), epistaxis (15), cardiac arrhythmia – bradycardia (17), syncope (17)
**Family-5**	1	*PDSS1*	28	Peripheral visual field loss	63	6/38 6/7.5	RP, myopic astigmatism, cataracts	Hypertension, myocardial infarction
**Family-6** **(ARRP195)**	1	*PDSS1*	37	Night blindness since childhood, glare sensitivity (37)	54	6/7.5 6/6	RP, myopic astigmatism, RE cataract	Premature birth (birth weight 1500 g), hemiplegia after birth (disappeared within an hour); osteomalacia. myocardial infarction (52)
Family -7 (GC26032)	1	*COQ2*	5	Night blindness, renal failure	35	6/9 6/9	RP	Renal failure
**Family-8** **(SRP1143)**	1	*COQ2*	39	Glare sensitivity	44	6/7.5 6/7.5	RP, myopic astigmatism, mild cataract, attenuated retinal vessels	
Family-9	3^c^	*COQ2*	ND	Night blindness, constricted visual field. RP diagnosis made later in life.	65	ND	RP	Renal transplantation (55), unknown cardiovascular disease
**Family-9** **(Proband)**			35	Reduced visual acuity	56	6/9.5 6/12	RP, CMO, posterior pole cataract, RE ERM	Lumbar herniated disc
**Family-10** **(GC20389)**	1	*COQ4*	29	Incidental finding during routine check-up (29). 6 years later started having vision problems: prolonged dark adaptation, followed by vision problems in the dark.	56	6/12 6/19	RP, hyperopia, attenuated retinal vessels	Hypertension, breast carcinoma (52)
**Family-11** **(GC21390)**	1	*COQ5*	49	Incidental finding during routine check-up (49). Prolonged dark adaptation, peripheral visual field loss	56	6/9 6/5	RP	Hypertension
Family-12	1	*COQ5*	5	Muscle weakness, followed by vision problems in the dark (14)	38	6/18 6/7.5	RP, nystagmus	Muscular weakness (hyposthenia), infantile appearance, hypertelorism, undeveloped fertile function,

Individuals in bold at the time of examination did not report extra-ocular signs and symptoms, which might be associated with primary CoQ10 deficiency.

*BCVA* best corrected visual acuity, *CMO* cystoid macular oedema, *ERM* epiretinal membrane, *LE* left eye, *ND* no data, *RE* right eye, *RD* retinal dystrophy, *RP* retinitis pigmentosa.

^a^Macular complications, such as cystoid macular oedema (CMO), epiretinal membrane (ERM), formation of a macular holes, are observed in patients with RP.

^b^Clinical data was obtained from details provided in Genomics England Research embassy platform.

^c^Only 2 out of 3 affected individuals from Family-9 were molecularly characterised.

### Molecular genetic analysis

Nine distinct variants in *PDSS1* (NM_014317.5, Table 1, Fig. 1), of which two, c.18 G > A, p.(Trp6*) and c.893dup, p.(Asn298Lysfs*11), were considered to be probable loss-of-function (LOF). Two variants were predicted to cause splice site disruption: c.722-2 A > G and c.468-25 A > G (Table 3). The variant c.589 A > G, p.(Lys197Glu) was identified in three affected individuals from three unrelated families (Table 1, Fig. 1) and in trans with a probable LOF or splice disrupting variant. This variant is present in 411/282764 alleles (MAF 0.001454) in gnomAD and a maximum allele frequency of 0.003499 is observed within the Latino/Admixed American population, with a single homozygote observed (gnomAD v3). The variant has 6 submissions to the ClinVar database (Benign (1 record), Likely benign (3 records), Variant of uncertain significance (2 records)). The Odds Ratio (OR) that *PDSS1* c.589 A > G, p.(Lys197Glu) is associated with disease is 108.34 (Fisher’s exact *p* = 4.699 × 10^−6^, 95% confidence interval: 20.47, 374.14. Supplementary Table 1). In light of the recurrence of this variant in trans with probable LOF variants, we considered this to be a candidate pathogenic genotype. No affected individual carried biallelic *PDSS1* null variants. Two patients carried biallelic missense or inframe indel variants that are considered to be possibly pathogenic at present (families 2 and 4, variants c.232 C > A, c.886 G > A, c.1118_1120dup).

**Table 3:** In silico prediction on aberrant splicing.

Gene	Splice variant	Position (GRCh37/hg19 build)	gnomAD (total allele number)	In silico prediction (confidence, intron^exon)
*PDSS1*	c.722-2 A > G	10-27012941-A-G	0.000003979 (1)	**Netgene2:**
				WT: (0.96, TTCTTTAT**A**G^GTGAATTTCT)
				M: (0.00, TTCTTTAT**G**G^GTGAATTTCT)
				Outcome: Canonical variant. Broken WT acceptor site and activation of cryptic acceptor site.
*PDSS1*	c.468-25 A > G	10-27009122-A-G	0.00001194 (3)	**Netgene2:** No splice defect
				**Splice AI:** Acceptor gain at -83 and acceptor loss at 0; Donor loss at +141
				Outcome: Intron retention
*COQ5*	c.682-7 T > G	12-120942793-A-C	0.0003007 (85)	**NetGene2:**
				WT: (0.68, TCT**T**GCTCAG^GCACTCCAGG)
				M: (0.28, TCT**G**GCTCAG^GCACTCCAGG)
				Outcome: Weakens acceptor site

*M* mutant, *WT* wild type.

Three different variants were identified in *COQ2* (NM_015697.9) with a previously reported variant c.683 A > G, p.(Asn228Ser) observed in all affected individuals from three unrelated families (Table 1). Haplotype analysis (data not shown) showed no shared single nucleotide variants (SNV) *in cis* with the variant in different families suggesting the variant to have arisen independently. Only one *COQ2* probable LOF variant, c.735 C > G, p.(Tyr245*) was identified, and this was in the individual observed in this study cohort to have childhood onset kidney disease.

One study subject was identified to carry two previously unreported missense variants in *COQ4* (NM_016035.5): c.692 G > A, p.(Cys231Tyr) and c.376 G > A, p.(Glu126Lys). No other candidate genotype was identified in any known disease gene following GS for this individual, therefore we consider the *COQ4* genotype to be possibly pathogenic in this case.

Three distinct variants were identified in the *COQ5* gene (NM_032314.4). A candidate non-canonical splice site variant (c.682-7 T > G) was identified in two unrelated individuals from families 11 and 12 in trans with a frameshift variant, c.933delC p.(Tyr311*), and missense, c.367 C > T p.(Arg123Trp), respectively.

All variants except *PDSS1* c.589 A > G, p.(Lys197Glu) and *COQ5* c.682-7 T > G were rare or absent from the gnomAD dataset (MAF <0.001, Table 1). The in silico algorithm, Mutation Taster, predicted all protein coding variants to be ‘disease causing’ with the exception of *PDSS1* c.589 A > G, p.(Lys197Glu), c.1118_1120dup, p.(Val373_Gln374insLeu) and *COQ2* c.338_341delinsG, p.(Val113_114delinsGly) which were predicted to be ‘polymorphisms’.

Multiple alignment of orthologues showed variable conservation of the protein across different species (Supplementary Fig. 2). Candidate splice site or splice region variants were rare in the gnomAD dataset and predicted to affect the canonical splice site by Netgene2 or SpliceAI (Table 3).

Haplotype analysis of three unrelated families showed a possible ancestral haplotype spanning about 398 kb flanking the *PDSS1* gene (Chr10:26,535,625 (rs187101868) to Chr10:26,933,928 (rs111256658)) in family 3. Family 6 shared the telomeric segment and family 5 shared the centromeric segment with two flanking SNPs shared between families 5 and 3 (Chr10:26,720,339 (rs116424900) to Chr10:26,933,928 (rs111256658)) and no flanking SNPs shared between families 5 and 6 (Supplementary Table 2).

The genome-wide filtering pipeline also identified biallelic rare variants in *MAST4* (proband, family 1), *SCML4* and *ZNF813* (proband, family 10) (Supplementary Tables 3–5). The variants in *PDSS1* and *COQ4* respectively were considered to be the most likely candidates based on the phenotypic similarity (pigmentary retinal changes have been previously reported in some affected individuals suffering from primary COQ10 deficiency) and lack of biological plausibility for other genes. For the proband from family 2, the virtual gene panel analysis identified variants in retinal genes, namely *USH2A* c.8555 T > C, p.(Leu2852Ser), *USH2A* c.14074 G > A, p.(Gly4692Arg), *RP1* c.4735 T > G, p.(Leu1579Val) and *COL4A1* c.4966 C > A, p.(Arg1656Ser). However, only one *USH2A* variant, c.4735 T > G, p.(Leu1579Val) was considered possibly associated with the phenotype, the second *USH2A* variant being classified as benign/likely benign in ClinVar. Thus, this case remained unsolved and underwent further analysis leading to the identification of biallelic *PDSS1* variants.

### Functional assessment of splice variants

**Fig. 3:**
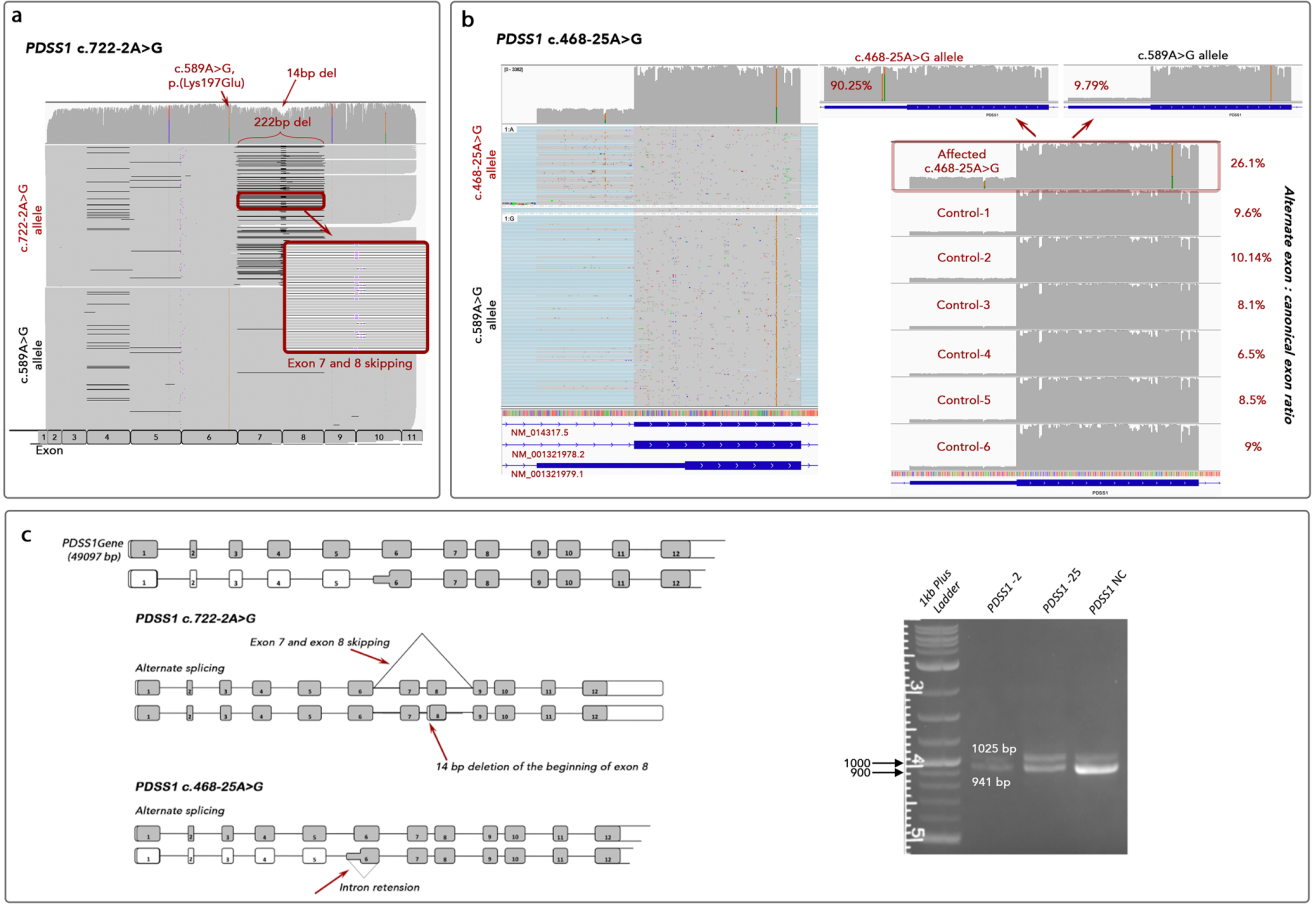
Detection of mis-splicing caused by variants in *PDSS1* by using ONT sequencing. **a** IGV visualisation of ONT single molecule sequencing of Individual family 3, II:2. Reads are aligned to the *PDSS1* transcript (PDSS1-201 ENST00000376215.10, NM_014317.5) and reads are grouped according to position c.589 A > G. Variant c.722-2 A > G leads to skipping of 222 bp (exon 7–8) or alternate acceptor usage in exon 8 (14 bp downstream). **b** IGV visualisation of ONT single molecule sequencing of individual from family 5, II:1. Reads are aligned to the human genome (build GRCh38) and *PDSS1* exon 6 is shown. Reads are grouped according to position c.589 A > G. Inclusion of the alternate exon (NM_001321979.1) is enriched on the c.468-25 A > G allele (90.25% versus 6.5-10.14% controls). **c** Schematic representation of cryptic splicing caused by c.722-2 A > G and c.468-25 A > G *PDSS1* variants and agarose gel analysis of RT-PCR reactions showing two transcripts in all samples. The 941 bp band corresponds to the canonical transcript and the 1025 bp band corresponds to the alternate exon 6 transcript. *PDSS1* NC shows a brighter band for the canonical transcript, while *PDSS1* -25 shows a reduced intensity. *PDSS1* -2 shows no additional transcripts. PDSS1 NC – normal control, PDSS1 -2 – individual with c.722-2 A > G. PDSS1 -25 – individual with c.468-25 A > G.

Three candidate splice variants, c.468-25 A > G and c.722-2 A > G in *PDSS1*, and c.682-7 T > G in *COQ5* were functionally investigated. RT-PCR of patient blood derived mRNA using oligonucleotide primers targeting *PDSS1* or *COQ5* (Supplementary Table 6) followed by agarose gel electrophoresis showed definite altered transcript length and mis-splicing only for the *COQ5* c.682-7 T > G variant. A reduced canonical transcript band intensity was observed in the sample from family 5, II:1 with the c.468-25 A > G variant in *PDSS1*. Subsequent Oxford Nanopore Technologies (ONT) single-molecule sequencing of RT-PCR reaction products revealed complex mis-splicing as a result of all three variants. As a result of *PDSS1* c.722-2 A > G disrupting the canonical intron 7 splice acceptor site, the altered splicing events were complete exon 7–8 skipping leading to an in-frame deletion of 72 codons (p.(Ala204_Ala277del)) or the use of an alternate splice acceptor site 14 bp downstream of the canonical acceptor site in exon 8 leading to deletion of 14 bp and a frameshift (p.(Gly241Alafs*6) (Fig. 3). The candidate variant *PDSS1* c.468-25 A > G was shown to lead to inclusion of an alternate noncanonical exon (Chr11:26,720,135-26,720,359) in the transcript or transcription of the alternate isoform of *PDSS1* (NM_001321979.1) with an alternate start codon in the alternate exon 6. For individual family 5, II:1 ONT sequencing of the *PDSS1* transcript enabled us to phase the two alleles on independent reads representing independent transcripts at the position of the missense variant (c.589 A > G). Comparing the read depth of the two alleles at this position on full-length reads (primer to primer, exon 1–11), it was clear that the c.589 A allele (representing the c.468-25 A > G variant allele) was underrepresented (27% vs 70%). The mean read depth for each nucleotide position of the alternate versus canonical exon (Chr11:26,720,135-26,720,217 and Chr11:26,720,218-26,720,359) for each allele (c.589 A and c.589 G) showed that 361/400 (90.25%) of the A allele and 159/1619 (9.79%) of the G allele included the alternate exon meaning that the c.468-25 A > G variant led to cryptic splicing in 90.25% of transcript reads. In six control RNA samples, the inclusion of the alternate exon was between 6.5 and 10.14%.

**Fig. 4:**
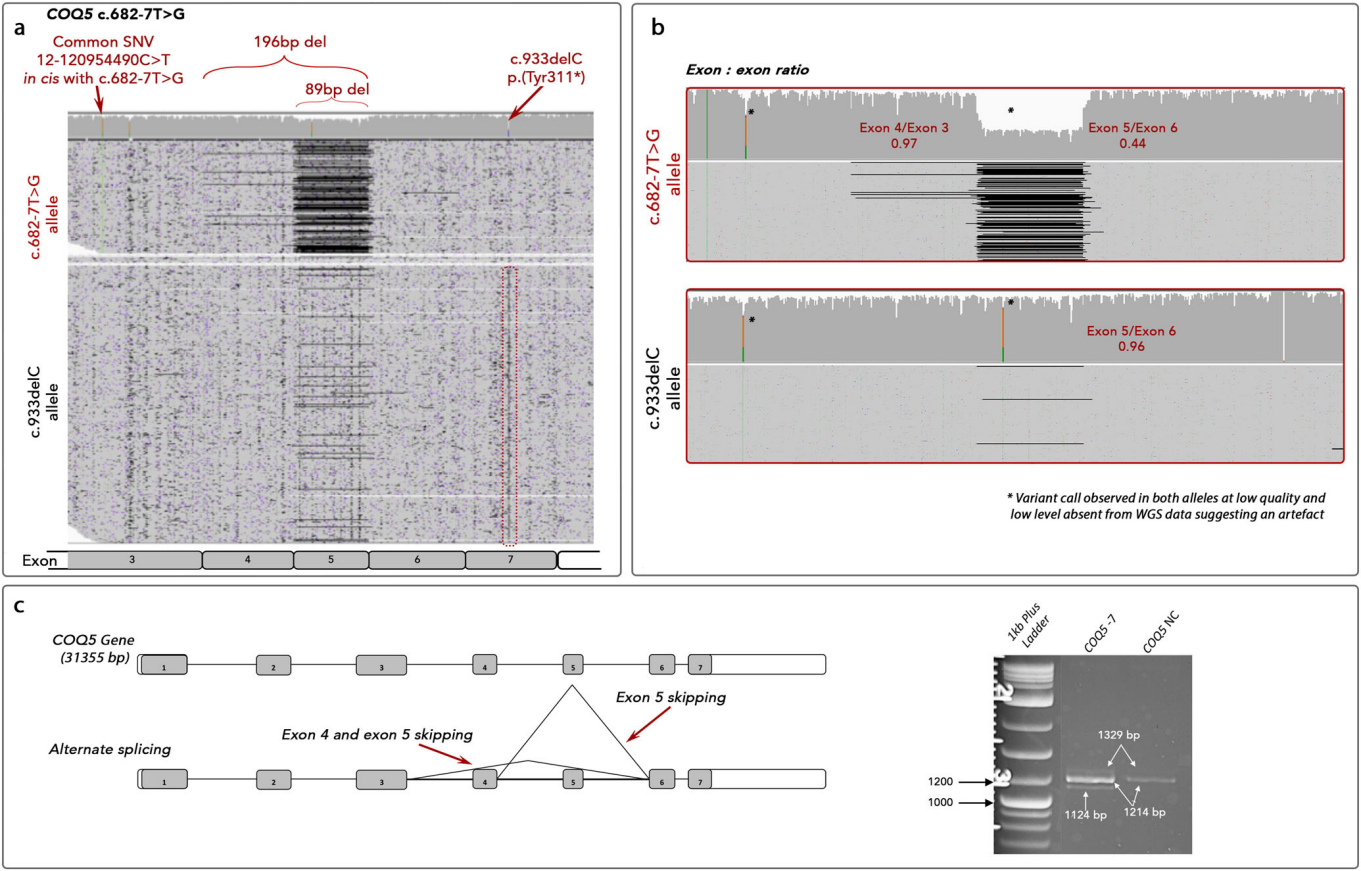
Detection of mis-splicing caused by c.682-7 T > G in *COQ5* by using ONT sequencing. **a** IGV visualisation of ONT single molecule sequencing of Individual family 11, II:1. Reads are aligned to the *COQ5* transcript (COQ5-201 ENST00000288532.11, NM_032314.4) and reads are grouped according to position c.933delC. Variant c.682-7 T > G leads to skipping of 89 bp (exon 5) and 196 bp (exon 4-5). **b** Split BAM files according to SNV at Chr12:120,954,490 to show the 56% coverage drop of exon 5 and low level exons 4–5 skipping on the c.682-7 T > G allele compared to low level of exon 5 skipping on the *trans* allele. **c** Schematic representation of cryptic splicing events caused by the c.682-7 T > G *COQ5* variant and agarose gel analysis of RT-PCR reactions showing alternate splicing. *Variant call observed in both alleles at low quality and low level absent from GS data suggesting an artefact.

One individual carried biallelic *COQ5* variants: c.682-7 T > G and c.933delC. One variant is predicted to disrupt splicing and the second predicted to be null. ONT sequencing of the full *COQ5* transcript from whole blood of individual family 11, II:1 showed skipping of exon 5 to be the predominant effect of the c.682-7 T > G variant leading to early termination and probable nonsense-mediated decay (p.(Gln230*)) (Fig. 4). In addition, exon 4–5 skipping was identified in a low proportion of reads (3%) leading to a reading-frame shift and early termination (p.(Leu193Phefs*27)). Genotyping of an additional observed SNV (Chr12:120,954,490 C > T) in the unaffected mother demonstrated this was *in cis* with c.682-7 T > G. This enabled phasing of the two transcript alleles showing that 56% of reads from the c.682-7 G allele showed exon 5 skippings compared to 4% of the *trans* allele. This suggests that a proportion of transcript is correctly spliced and may lead to mature protein, therefore, suggesting this variant is not a complete LOF allele. A low level of mis-spicing was observed on the *trans* allele (4%) and in control samples (data not shown) suggesting there may be a basal level of alternate splicing at this position leading to non-expressed transcripts but possibly also a low level of miscalling at the Chr12:120,954,490 position in our data leading to a low proportion of incorrect phasing.

### Biochemical studies

Human plasma CoQ_10_ levels in 8 affected individuals and 3 heterozygotes were within the normal range (reference range 227- 1432 nmol/L, Supplementary Table 7). Individual family 7, II:1 carrying biallelic variants in *COQ2* in association with syndromic disease had severely decreased plasma CoQ_10_ concentration of 39.33 nmol/L.

## Discussion

This study reports clinical and genetic findings in a cohort of patients carrying biallelic candidate pathogenic variants in CoQ_10_ biosynthesis pathway genes and diagnosed with retinitis pigmentosa with or without extraocular disease (hearing loss, cardiac disease, kidney disease). We demonstrate that such gene defects represent a mechanism of non-syndromic retinopathy in six individuals (family 6, 8, 9 (one individual), 10, 11, see Table 2). In this case series, all but one individual tested had unaltered plasma CoQ_10_ levels which may suggest the effect of these CoQ_10_ biosynthesis pathway defects are mild although it is recognised that plasma levels are a poor indicator of intracellular coenzyme Q10 deficiency. In addition, we demonstrate the utility of ONT sequencing to characterise in detail the aberrant splicing consequence of non-canonical and canonical splice site variants where simple agarose gel analysis of RT-PCR products was not informative meaning that Sanger sequencing could not easily be performed. Single-molecule sequencing has the advantage of producing a single read for every molecule of RT-PCR amplicon that is captured in the library preparation rather than an accumulation of signal that must reach a threshold of detection (agarose gel band) or a mix of amplicon sequencing (Sanger sequencing of RT-PCR reactions).

To date, biallelic variants in CoQ_10_ biosynthesis pathway genes have been reported to cause primary coenzyme Q10 deficiency, usually associated with multisystemic involvement and only recently demonstrated to show retinal involvement^5–7^. Nonetheless, a recent study reported a nephropathy and retinopathy without neurologic manifestations in a family carrying biallelic *COQ2* variants^11^. In our patient cohort, candidate biallelic genotypes were identified in four CoQ_10_ biosynthesis pathway genes: *PDSS1*, *COQ2*, *COQ4* and *COQ5*.

Previous reports describing the clinical features of *PDSS1*-disease highlighted optic atrophy and sensorineural hearing loss as major features in all cases^16–18^. Intriguingly, none of those reports indicated pigmentary retinal changes on ophthalmological assessment, and none of our affected individuals showed signs and symptoms of optic atrophy. It is possible that the retinopathy had yet to manifest with a mean of age of examination for previous reported cases of 14 years. In our study, the mean of age when visual symptoms were reported by individuals carrying candidate pathogenic *PDSS1* genotypes was 28 years with some individuals not reporting symptoms (until directly questioned) due to preserved central vision. Also, retinal changes can be very mild, and may be missed in the absence of a detailed fundus examination and multimodal imaging.

It is an emerging phenomenon that unbiased sequencing investigations reveal mild or hypomorphic missense alleles leading to non-syndromic retinopathy or an attenuated syndromic phenotype with predominant retinal involvement in previously known genes associated with syndromic disease^14,19^. The *PDSS1* c.589 A > G p.(Lys197Glu) variant was seen in three unrelated individuals in our study cohort. Although previously classified as likely benign, we observed this in trans with a probable LOF variant in all cases, which may indicate that the variant may be mild and only pathogenic when in trans with a null allele, similar to the situation in *ABCA4*-retinopathy where the common c.5603 A > T p.(Asn1868Ile) variant is only thought to result in disease in trans with a severe counter-allele^20^. Nevertheless, we demonstrated a likely ancestral haplotype harbouring this variant and therefore cannot exclude the possibility that other variants could account for or modify the pathogenicity of the allele compared to potentially different haplotypes observed in the gnomAD dataset.

Three distinct variants were identified in the *COQ5* gene. To our knowledge, Mendelian disease associated with single nucleotide variants in this gene have not been reported before. Our analysis points towards a definitive relationship between reduced *COQ5* function and the retinopathy in two unrelated families. It is important to note that a previous report showed a homozygous partial tandem duplication of the 3’ end of the *COQ5* gene in 3 affected siblings with varying degree of cerebellar ataxia, encephalopathy, generalized tonic-clonic seizures, and cognitive disability^21^.

Due to limitations caused by the COVID-19 pandemic, we were not able to obtain muscle biopsy or fibroblast samples for CoQ_10_ levels measurements. However, we collected blood samples to study plasma CoQ_10_ levels as previously described^22^. This indicated severe reduction of CoQ_10_ level in one individual, who had kidney and retinal disease. A borderline reduction was observed in one additional individual from family 6, II:1 (195.45, reference range 227–1432 nmol/L) with the remaining individuals having normal CoQ_10_ levels. Without available fibroblasts or muscle biopsy, interpreting these findings is difficult since plasma CoQ_10_ measurements may not truly represent the intracellular CoQ_10_ level generated by the CoQ_10_ biosynthesis pathway as most individuals obtain sufficient levels of CoQ_10_ through a balanced diet, therefore it is difficult to draw any conclusion from the normal levels observed in all but one individual^22^. None of the study participants were on CoQ_10_ supplementation. Hence, CoQ_10_ intracellular levels measured in muscle biopsy or fibroblasts would have been attempted if possible, to better reflect the enzymatically produced CoQ_10_ levels.

CoQ10 analogues are a promising approach for the management of patients with primary mitochondrial diseases and it has been shown that patients with primary CoQ_10_ deficiency are responsive to CoQ_10_ supplementation^23^. Importantly, it has been demonstrated that aging leads to reduced CoQ_10_ levels in the retina^24^. Such decline may lead to reduction of ATP production within the retina as well as a decrease in antioxidant capacity, which may contribute to the progression of retinal degeneration. Notably, lower total antioxidant capacity has been demonstrated in the aqueous humour and sera from patients with RP^25^. Thus, reduced capacity for ATP production consequent upon CoQ_10_ biosynthesis gene defects may represent a mechanism for RP in these cases.

However, the major limitation of CoQ_10_ supplementation therapy is failure of CoQ_10_ to cross the blood-brain barrier in patients diagnosed with mitochondrial eye diseases. Yet, idebenone, a newer generation quinone analogue is able to penetrate central nervous system tissues and improve energy supply by bypassing the defective mitochondrial respiratory chain complex I and transporting electrons directly to complex III. Treatment trials have provided convincing evidence that oral idebenone has an effect in preventing further vision loss and even vision recovery in patients with another mitochondrial eye disorder - Leber hereditary optic neuropathy (LHON)^26,27^. In view of the non-syndromic retinal phenotype observed in our study, it would be useful in the future to consider an alternative to CoQ_10_ supplementation.

In summary, we characterise gene defects of the CoQ_10_ biosynthesis pathway that lead to a previously unreported association with non-syndromic retinopathy. GS is a powerful tool allowing scientists to reach a likely molecular diagnosis in previously unsolved cases by interrogation of all genes and intronic regions. Using RT-PCR and ONT sequencing we were able to characterise in detail the effect of splice altering variants where simple agarose gel analysis of RT-PCR products was not informative. Coenzyme Q10 biosynthesis pathway genes should be considered in ocular genetic panels including those investigating optic atrophy and retinopathy.

## Methods

### Study cohort

Patients and families were identified from the genetically unsolved cohort of individuals having had prior investigation for variants in genes known to harbour pathogenic variants that cause IRD. Six families were identified in the inherited eye disease clinics at Moorfields Eye Hospital NHS Foundation Trust (London, UK), with one additional UK individual identified via the UK’s 100,000 Genomes Project (100KGP). Five families were identified through the European Retinal Disease Consortium (ERDC, https://www.erdc.info) with GS performed at Radboud University Medical Centre, Nijmegen, the Netherlands, University Eye Hospital Tübingen, Germany and exome sequencing (ES) at the Institute of Molecular and Clinical Ophthalmology Basel (IOB), Switzerland.

The clinical and genetic data of individuals with candidate biallelic variants in CoQ_10_ pathway genes were reviewed to establish the ocular and extraocular phenotypic spectrum where available. After candidate variants were identified in coenzyme Q10 pathway genes, blood samples were collected using serum separation tubes (SST) from 9 affected individuals and 3 heterozygote relatives to quantify the circulating CoQ_10_ level in plasma. Written informed consent was obtained for all study participants. The study adhered to the tenets of the Declaration of Helsinki and all contributing study centres had the relevant local and national research ethics committee approvals: Moorfields Eye Hospital NHS Foundation Trust and the Northwest London Research Ethics Committee (REC 12/LO/0141), the Radboud University Medical Centre, Nijmegen, The Netherlands, the Rotterdam Eye Hospital, Rotterdam, The Netherlands (MEC-2010-359; OZR protocol nr. 2009-32), the Ethics Committee of the University of Tübingen, Germany (project no. 116/2015BO, as of 15 June 2018) and the Institute of Molecular and Clinical Ophthalmology Basel (IOB), Switzerland (Ethikkommission Nordewest- and Zentralschweiz (EKNZ), # 2019-01660). For patients and relatives recruited for the 100KGP, informed consent for GS was obtained in accordance with approval from the HRA committee East of England-Cambridge south (REC 14/EE/1112).

### Clinical phenotyping

Medical records were reviewed for 11/12 affected individuals following the identification of candidate pathogenic variants in the *PDSS1*, *COQ2*, *COQ4* and *COQ5* genes. All affected individuals underwent a comprehensive ophthalmological examination during the initial diagnostic work up or following the molecular investigations.

### Molecular genetics

GS was performed as part of the National Institute for Health Research BioResource rare-disease project (NIHR-RD) and 100KGP for families 1–4, 7, 10–11^28,29^. These two studies included 722 individuals with IRD (NIHR-RD) and 1437 probands with rod-cone dystrophy (100KGP). All affected individuals underwent prior clinical variant interrogation using the Panelapp Retinal disorders gene panel to identify pathogenic or likely pathogenic genotypes in genes already associated with IRD^28,30^. Patients who were negative following this pipeline underwent further investigation to identify candidate biallelic genotypes in CoQ_10_ biosynthesis pathway genes (*PDSS1, PDSS2, COQ2, COQ3, COQ4, COQ5, COQ6, COQ7, COQ8A, COQ8B, COQ9, COQ10A*, and *COQ10B*): rare variants (minor allele frequency, MAF < 0.001) were identified, including protein altering, splice site, potential cryptic splice altering intronic and structural variants. GS for families 5 and 12 were performed by BGI (Hong Kong, China) on a BGISeq500 using a 2 × 100 bp paired-end reads, with a minimal median coverage per genome of 30-fold and processed as previously described^31^. Briefly, Burrows‐Wheeler Aligner (v0.7.13) was used to map GS data to the human genome (hg19/GRCh37) and variant calling was carried out using xAtlas V.0.1 and annotated using the Variant Effect Predictor (VEP V.91) and Gencode V.34lift37 basic gene annotations. A subsequent CoQ_10_ pathway specific variant analysis was performed after an initial GS variant prioritization for IRD-associated genes remained negative. For families 6 and 8, GS was conducted in a routine diagnostic context as part of a cohort of approximately 1300 affected individuals^32^. Briefly, GS (2 × 150 bp paired-end reads) was performed on an Illumina platform (NovaSeq6000) and bioinformatic processing of generated sequences, variant calling and annotation was performed using the megSAP pipeline (https://github.com/imgag/megSAP). Clinical variant prioritization was done according to an in-house standard operating procedure and included different filtering steps of detected SNV and structural variants for e.g., allele frequency (MAF < 0.001) and predicted impact on protein function or pre-mRNA splicing. DNA from the proband of family 9 was processed by ES. Exome libraries (Agilent SureSelect Human All Exon V6 kit; Agilent Technologies) were sequenced on an Illumina Novaseq 6000 at the Novogene Co. Ltd. in Cambridge, UK. Raw reads were mapped to the human reference genome (hg19/GRCh37) using BWA (v0.7.17). Next, Picard (version 2.14.0-SNAPSHOT) was used to remove duplicate reads and Genome Analysis Toolkit (GATK) (v4.1.4.1) was used to perform base quality score recalibration on both single-nucleotide variants and insertion–deletions. A VCF file with the variants was generated by HaplotypeCaller (GATK, v4.1.4.1). Variants were then analysed as previously described^33^.

### Variant phasing

Where possible (trio or family GS analysis), variants were phased by interrogation of the GS data (families 1, 3, 4, 7). Variants that were in close proximity to each other (within the same read pairs), could be phased by interrogating the individual paired-end read data using Integrative Genomics Viewer (IGV)^34^. Bidirectional Sanger sequencing using standard reagents and protocols was performed to confirm segregation in available relatives in families 6, 8, 9 and 11) (primers can be found in Supplementary Table 6). Since no family members were available for phasing in family 2 and family 5, the mRNA transcript of *PDSS1* was sequenced using an ONT single-molecule sequencing approach. Briefly, total RNA was purified from PAXgene stabilised whole blood and reverse transcribed using random hexamers and SuperScript IV reverse transcriptase (Invitrogen). Polymerase chain reaction was performed for a 941 bp amplicon of the *PDSS1* transcript (exon 1–11). Agencourt AMPure XP beads (Beckman Coulter) were used to purify the amplicon, followed by library preparation using the ONT Genomic DNA by Ligation (SQK-LSK109) protocol and sequencing on a flongle flow-cell for approximately 12 h. Fast5 raw data files were basecalled and aligned using the Guppy v 3.2.10 and Minimap2^35^ software packages and BAM files generated using SAMtools^36^. Variants were phased by interrogating the individual long reads using IGV^34^. Neither familial DNA samples nor PAXgene RNA were available to establish phase in families 10 and 12. All agarose gels presented in the figures derive from a single experiment.

### Bioinformatics

The Genome Aggregation Database (gnomAD, https://gnomad.broadinstitute.org) was used to assess the frequency of variants in the general population. To evaluate the likely impact of missense variants on protein function, in silico analysis was performed using the Mutation Taster (http://www.mutationtaster.org/) predictive algorithm. Candidate variants were annotated based on the ACMG guidelines using the VarSome prediction tool (accessed August 2021)^37^. The evolutionary conservation of the affected amino acid residues across orthologues was assessed using Uniprot (https://www.uniprot.org). Predicted effect on pre-mRNA splicing was investigated with the in silico splice tools Netgene2 (http://www.cbs.dtu.dk/services/NetGene2/). In addition, SpliceAI (https://github.com/Illumina/SpliceAI) was used to assess the *PDSS1* c.468-25 A > G variant. At least one delta-score above the threshold of 0.01 for gain or loss in a splice donor or acceptor site was used for a putative pathogenic effect. The input sequence for SpliceAI analysis included 1000 bp up and downstream of the variant. Automap was used to detect runs of homozygosity on data from ES^38^.

### Potential splice variant characterisation

Variants predicted to have an altered splice effect, were investigated by RT-PCR on patient derived total RNA and ONT-single molecule sequencing to characterise resultant transcripts. Reads were mapped to either the human reference genome (GRCh38) or cDNA sequences: *PDSS1* (PDSS1-201 ENST00000376215.10) or *COQ5* (COQ5-201 ENST00000288532.11) using Minimap2^35^. BAM files were generated using SAMtools and visualised with IGV^34^. Control RNA from unrelated individuals were used to evaluate the alterations.

### Haplotype analysis

The haplotypes of recurrently identified variants were investigated using the familial GS data. SNV up to approximately 250 kb up and down-stream of the gene of interest were phased to determine those *in cis* with the identified variant. SNV with MAF < 0.1 in the gnomAD dataset were considered to be informative for phasing.

### Biochemical studies

Human plasma specimens were used to assess CoQ_10_ levels in 9 affected individuals and 3 heterozygotes. The CoQ_10_ status of the human plasma samples was determined using high pressure liquid chromatography with UV detection at 275 nm as previously described by the method of Duncan et al. (2005)^22^. CoQ10 was extracted from plasma (100 μl) using a solvent of hexane: ethanol (5:2, v/v). The upper hexane layer was retained for analysis and evaporated under N_2_ gas. After re-suspension in HPLC grade ethanol, separation was achieved using a reverse phase C18 column and a mobile phase composed of methanol/ethanol/perchloric (700:300:1.2) at a flow rate of 0.7 ml/min. Plasma CoQ_10_ status was determined by comparison to known standard of CoQ_10_. Dipropoxy-CoQ_10_ was used as the internal standard in this HPLC method. The results were expressed in nmol/L. Tentative reference range for CoQ_10_ levels in human plasma was considered as 227–1432 nmol/L.

### Statistical analysis

To investigate if the recurrent *PDSS1* c.589 A > G, p.(Lys197Glu) variant is associated with disease in the *PDSS1-*disease cohort, a Fisher's exact test was performed and OR was calculated using RStudio (Version 1.2.1093). Statistical significance was considered when *P* ≤ 0.05.

### Reporting summary

Further information on research design is available in the Nature Research Reporting Summary linked to this article.

## Supplementary information

Reporting Summary

Supplementary Material

## Data Availability

Further details of the GS and ES data presented in the study are available via direct contact with the corresponding author. Data sharing is possible for non-commercial use via an institutional review board approved collaborator agreement. Data accessibility information for the 100 KGP is available online (www.genomicsengland.co.uk/join-a-gecip-domain). ES and GS data could not be deposited in any repository due to ethical restrictions and data sharing not being part of the patient consent.

## Code availability

Packages and software used are described in the “Methods” section.
