# Supplementary material for: Biallelic variants in coenzyme Q10 biosynthesis pathway genes cause a retinitis pigmentosa phenotype
Source: NPJ Genom Med. 2022 Oct 20;7:60. doi: 10.1038/s41525-022-00330-z (PMC9581764; doi:10.1038/s41525-022-00330-z)
Supplement: Supplementary file 1 — Reporting Summary [file 41525_2022_330_MOESM1_ESM.pdf]

nature portfolio

Corresponding author(s): Gavin Arno, Neringa Jurkute

Last updated by author(s): Aug 23, 2022

Reporting Summary

Nature Portfolio wishes to improve the reproducibility of the work that we publish. This form provides structure for consistency and transparency in reporting. For further information on Nature Portfolio policies, see our Editorial Policies and the Editorial Policy Checklist.

Statistics

For all statistical analyses, confirm that the following items are present in the figure legend, table legend, main text, or Methods section.

n/a	Confirmed	
☒	☐	The exact sample size (*n*) for each experimental group/condition, given as a discrete number and unit of measurement
☒	☐	A statement on whether measurements were taken from distinct samples or whether the same sample was measured repeatedly
☐	☒	The statistical test(s) used AND whether they are one- or two-sided<br>*Only common tests should be described solely by name; describe more complex techniques in the Methods section.*
☒	☐	A description of all covariates tested
☒	☐	A description of any assumptions or corrections, such as tests of normality and adjustment for multiple comparisons
☒	☐	A full description of the statistical parameters including central tendency (e.g. means) or other basic estimates (e.g. regression coefficient) AND variation (e.g. standard deviation) or associated estimates of uncertainty (e.g. confidence intervals)
☒	☐	For null hypothesis testing, the test statistic (e.g. *F*, *t*, *r*) with confidence intervals, effect sizes, degrees of freedom and *P* value noted<br>*Give P values as exact values whenever suitable.*
☒	☐	For Bayesian analysis, information on the choice of priors and Markov chain Monte Carlo settings
☒	☐	For hierarchical and complex designs, identification of the appropriate level for tests and full reporting of outcomes
☒	☐	Estimates of effect sizes (e.g. Cohen's *d*, Pearson's *r*), indicating how they were calculated

Our web collection on statistics for biologists contains articles on many of the points above.

Software and code

Policy information about availability of computer code

Data collection

Provide a description of all commercial, open source and custom code used to collect the data in this study, specifying the version used OR state that no software was used.

Data analysis	Burrows-Wheeler Aligner (Version???)<br>xAtlas V.0.1<br>Variant Effect Predictor (VEP V.91)<br>megSAP pipeline (https://github.com/imgag/megSAP)<br>Novoalign software (V3.08.00, Novocraft Technologies)<br>Picard (version 2.14.0-SNAPSHOT)<br>Genome Analysis Toolkit (GATK) (version 3.8)<br>HaplotypeCaller (version?????)<br>Integrative Genomics Viewer (IGV, v2.5.0).<br>Oxford Nanopore technologies Guppy v 3.2.10<br>Minimap2 v2.17<br>SAMtools v1.10-99-g778aecb<br>gnomAD, https://gnomad.broadinstitute.org<br>Mutation Taster, http://www.mutationtaster.org/<br>VarSome prediction tool (accessed August 2021)<br>Netgene2, http://www.cbs.dtu.dk/services/NetGene2/<br>SpliceAI, https://github.com/Illumina/SpliceAI<br>RStudio (Version 1.2.1093)

For manuscripts utilizing custom algorithms or software that are central to the research but not yet described in published literature, software must be made available to editors and reviewers. We strongly encourage code deposition in a community repository (e.g. GitHub). See the Nature Portfolio guidelines for submitting code & software for further information.

Data

Policy information about availability of data

All manuscripts must include a data availability statement. This statement should provide the following information, where applicable:

- Accession codes, unique identifiers, or web links for publicly available datasets
- A description of any restrictions on data availability
- For clinical datasets or third party data, please ensure that the statement adheres to our policy

Further details of the GS and ES data presented in the study are available via direct contact with the corresponding author. Data accessibility information for the 100KGP is available online (www.genomicsengland.co.uk/join-a-gecip-domain). ES and GS data could not be deposited in any repository due to ethical restrictions and data sharing not being part of the patient consent.

Human research participants

Policy information about studies involving human research participants and Sex and Gender in Research.

Reporting on sex and gender	n/a
Population characteristics	n/a
Recruitment	Patients and families were identified from the genetically unsolved cohort of individuals having had prior investigation for variants in genes known to harbour pathogenic variants that cause IRD. Six families were identified in the inherited eye disease clinics at Moorfields Eye Hospital NHS Foundation Trust (London, UK), with one additional UK individual identified via the UK's 100,000 Genomes Project (100KGP). Five families were identified through the European Retinal Disease Consortium (ERDC, https://www.erdc.info) with GS performed at Radboud University Medical Center, Nijmegen, the Netherlands, University Eye Hospital Tübingen, Germany and exome sequencing (ES) at the Institute of Molecular and Clinical Ophthalmology Basel (IOB), Switzerland.
Ethics oversight	Written informed consent was obtained. The study adhered to the tenets of the Declaration of Helsinki and all contributing study centres had the relevant local and national research ethics committee approvals: Moorfields Eye Hospital NHS Foundation Trust and the Northwest London Research Ethics Committee (REC 12/LO/0141), the Radboud University Medical Center, Nijmegen, The Netherlands, the Rotterdam Eye Hospital, Rotterdam, The Netherlands (MEC-2010-359; OZR protocol nr. 2009-32), the Ethics Committee of the University of Tübingen, Germany (project no. 116/2015BO, as of 15 June 2018) and the Institute of Molecular and Clinical Ophthalmology Basel (IOB), Switzerland (Ethikkommission Nordewest- and Zentralschweiz (EKNZ), # 2019-01660). Written informed consent was obtained from study subjects. For patients and relatives recruited for the 100KGP, informed consent for GS was obtained in accordance with approval from the HRA committee East of England-Cambridge south (REC 14/EE/1112).

Note that full information on the approval of the study protocol must also be provided in the manuscript.

Field-specific reporting

Please select the one below that is the best fit for your research. If you are not sure, read the appropriate sections before making your selection.

☒ Life sciences ☐ Behavioural & social sciences ☐ Ecological, evolutionary & environmental sciences

For a reference copy of the document with all sections, see nature.com/documents/nr-reporting-summary-flat.pdf

Life sciences study design

All studies must disclose on these points even when the disclosure is negative.

Sample size	Patients were selected for study from the available cohorts of unsolved GS or ES analysed IRD patients. The selection criterion was to harbour a rare biallelic genotype in a CoQ10 pathway gene.
Data exclusions	No data were excluded
Replication	n/a
Randomization	n/a
Blinding	n/a

Reporting for specific materials, systems and methods

We require information from authors about some types of materials, experimental systems and methods used in many studies. Here, indicate whether each material, system or method listed is relevant to your study. If you are not sure if a list item applies to your research, read the appropriate section before selecting a response.

Materials & experimental systems

n/a	Involved in the study	
☒	☐	Antibodies
☒	☐	Eukaryotic cell lines
☒	☐	Palaeontology and archaeology
☒	☐	Animals and other organisms
☐	☒	Clinical data
☒	☐	Dual use research of concern

Methods

n/a	Involved in the study	
☒	☐	ChIP-seq
☒	☐	Flow cytometry
☒	☐	MRI-based neuroimaging

Clinical data

Policy information about clinical studies

All manuscripts should comply with the ICMJE guidelines for publication of clinical research and a completed CONSORT checklist must be included with all submissions.

Clinical trial registration	n/a
Study protocol	n/a
Data collection	clinical data was collected from patient records at the referring centre
Outcomes	n/a
