# Supplementary material for: Biallelic variants in coenzyme Q10 biosynthesis pathway genes cause a retinitis pigmentosa phenotype
Source: NPJ Genom Med. 2022 Oct 20;7:60. doi: 10.1038/s41525-022-00330-z (PMC9581764; doi:10.1038/s41525-022-00330-z)
Supplement: Supplementary file 2 — Supplementary Material [file 41525_2022_330_MOESM2_ESM.docx]

Supplementary Table 1. Recurrent *PDSS1* c.589A>G, p.(Lys197Glu) variant allele frequency within the *PDSS1*-associated disease cohort and the general population.

| c.589A>G, p.(Lys197Glu) variant frequency | Observed | Not observed | Total allele frequency |
| --- | --- | --- | --- |
| PDSS1-associated disease (current study and previous reports) | 3 | 19 | 22 |
|  |  |  |  |
| gnomAD v2 allele frequency | 411 | 282353 | 282764 |
| Total allele frequency | 414 | 282372 | 282786 |
| Fisher’s exact test p-value | 4.7x10-6 |  |  |
| Odds ratio  Confidence interval | 108.3  20.5-374.1 |  |  |

Supplementary Table 2. Haplotype reconstruction for individuals carrying candidate *PDSS1* variant c.589A>G, p.(Lys197Glu).

|  |  |  |  | Haplotype | | | Genotype | | | | |  |
| --- | --- | --- | --- | --- | --- | --- | --- | --- | --- | --- | --- | --- |
| rs number | Position (Build38) | gnomAD MAF | Reference allele | Family-3, II:2 (GC35315) | Family-5, II:1 | Family-6, II:1 (ARRP195) | Family-3*, I:1 (GC35315) | Family-3*, II:2 (GC35315) | Family-3, I:2 (GC35315) | Family-5*, II:1 | Family-6*, II:1 (ARRP195) |  |
|  |  |  |  |  |  |  |  |  |  |  |  |  |
| rs117577922 | chr10:26263890 | 0.01534 | G | T | G | G | G/T | G/T | G/G | G/G | G/G |  |
| rs116871845 | chr10:26265146 | 0.0147 | C | T | C | C | C/T | C/T | C/C | C/C | C/C |  |
| rs8190731 | chr10:26274024 | 0.008339 | C | T | C | C | C/T | C/T | C/C | C/C | C/C |  |
| rs777036315 | chr10:26397715 | 0.00005944 | T | C | T | T | T/C | T/C | T/T | T/T | T/T |  |
| rs117257515 | chr10:26514025 | 0.01227 | C | T | C | C | C/T | C/T | C/C | C/C | C/C |  |
| rs187101868 | chr10:26535625 | 0.002831 | T | C | T | C | T/C | T/C | T/T | T/T | T/C |  |
| rs145010792 | chr10:26550063 | 0.03022 | C | G | C | G | C/G | C/G | C/C | C/C | C/G |  |
| rs549881251 | chr10:26634779 | 0.01101 | C | A | C | A | C/A | C/A | C/C | C/C | C/A |  |
| rs184987424 | chr10:26634780 | 0.01002 | G | T | G | T | G/T | G/T | G/G | G/G | G/T |  |
| rs61838905 | chr10:26640734 | 0.003427 | G | A | G | A | G/A | G/A | G/G | G/G | G/A |  |
| rs61838926 | chr10:26663970 | 0.009071 | A | G | A | G | A/G | A/G | A/A | A/A | A/G |  |
| rs61838928 | chr10:26671614 | 0.03754 | G | T | G | T | G/T | G/T | G/G | G/G | G/T |  |
| rs117380133 | chr10:26675433 | 0.009093 | G | A | G | A | G/A | G/A | G/G | G/G | G/A |  |
| rs186843926 | chr10:26713970 | 0.00872 | G | A | G | A | G/A | G/A | G/G | G/G | G/A |  |
| **rs116424900** | **chr10:26720339** | **0.001793** | **A** | **G** | **G** | **G** | **A/G** | **A/G** | **A/A** | **A/G** | **A/G** |  |
| rs776918911 | chr10:26864245 | 0.00007907 | T | TT | TT | T | T/TT | T/TT | T/T | T/TT | T/T |  |
| rs111256658 | chr10:26933928 | 0.04089 | A | G | G | A | A/G | A/G | A/A | A/G | A/A |  |
| rs149035234 | chr10:27042557 | 0.0338 | T | C | T | T | T/C | T/C | T/T | T/T | T/T |  |
| rs200736107 | chr10:27248127 | 7.754E-06 | C | G | C | C | C/G | C/G | C/C | C/C | C/C |  |

Haplotypes were reconstructed using GS datasets (100KGP study). Candidate variant (rs116424900) c.589A>G, p.(Lys197Glu) is in bold. Shaded haplotype is shared.

* individuals are heterozygotes for candidate c.589A>G, p.(Lys197Glu) variant.

Supplementary Table 3. Rare variant filtering pipeline for individuals identified through Genomics England 100KGP.

| Filtering pipeline |  | Family-1  (GC3635) | Family-3  (GC35315) | Family-7  (GC26032) |
| --- | --- | --- | --- | --- |
|  | Total variants | 5537226 | 5557329 | 5395563 |
|  | Primary analysis (Clinical pipeline) | | | |
|  | Virtual panels applied | Negative Posterior segment abnormalities 1.86 Congenital hearing impairment 1.47 Mitochondrial disorders 1.68 | Negative Retinal disorders 1.92 Optic neuropathy 1.25 | Negative Retinal disorders 1.92 |
|  | CNV (>10kb high quality calls) | None reported | None reported | None reported |
|  | Short tandem repeats only for specific loci defines in the virtual panel(s) applied | None reported | None reported | None reported |
|  | Candidate variants | Not identified | Not identified | Not identified |
|  | Outcome after clinical pipeline | Unsolved | Unsolved | Unsolved |
|  | Secondary analysis (Research pipeline) revealing number of alleles | | | |
|  | gnomAD MAF<0.001 | 647459 | 935432 | 608940 |
|  | LOF/missense | 2429 | 1955 | 1837 |
|  | RD38 MAF<0.001 | 234 | 162 | 231 |
|  | Tier 1 | 0 | 0 | 0 |
|  | Tier 2 | 0 | 0 | 0 |
|  | Tier 3 | 236 | 55 | 332 |
|  | Biallelic | 48 | 24 | 71 |
|  | LOF/missense | 18 | 11 | 26 |
|  | Rare in GE dataset (<10 alleles) | 6 | 6 | 3 |
|  | Genes remaining with biallelic variants (from the analysis above) | | | |
|  | Genes remaining with biallelic variants (see supplementary table 3) | *MAST4* and *PDSS1* | *PDSS1*, *PNPLA2* and *MUC12* | *COQ2* |
| Tiered variants are rare variants that segregate with disease.  Tier 1 - includes high impact variants, such as stop-gained, stop-lost, start-lost, splice donor, splice acceptor, frameshift, transcript ablation, de novo in a monoallelic gene, within a curated gene list with a sufficient evidence associating them with the patients phenotype. Tier 2 - includes moderate impact variants, such as missense, splice region variant, in-frame insertion, in-frame deletion, transcript amplification, incomplete terminator codon, within a curated gene list with a sufficient evidence associating them with the patients phenotype. Tier 3 - includes high and moderate impact variants outside the curated gene list associated with the patients phenotype. | | | | |

Supplementary Table 4. Rare variant filtering pipeline for individuals identified through NIHR-RD study.

| Filtering pipeline |  | Family-10  (GC20389) | Family-11  (GC21390) |
| --- | --- | --- | --- |
|  | Total rare* | 4920 | 941 |
|  | gnomAD MAF<0.001 | 1109 | 319 |
|  | Impact (High/moderate) | 725 | 209 |
|  | Impact on canonical transcript | 503 | 149 |
|  | Biallelic | 65 | 6 |
|  | gnomAD MAF<0.001 | 23 | - |
|  | Genes remaining with  biallelic variants (see supplementary table 3) | 16 | - |

* rare variants in NIHR-RD dataset are the ones with MAF<0.001 within the NIHR-RD and ExAc datasets.

Supplementary Table 5. Candidate and additional rare variants identified in affected individuals.

| **Proband** | **Symbol** | **Position** | **Build** | **gnomAD  v3 for Build38 v2 for Build37** | **Effect** | **GE dataset  (no of families)** | **Zygosity** | **Associated disease** | **OMIM** | **Inheritance** | **Comment** |
| --- | --- | --- | --- | --- | --- | --- | --- | --- | --- | --- | --- |
| Family-1  (GC3635) | *MAST4* | 5:67165221 TCTC>T | 38 | 0.001068 | Inframe deletion | 2 | Het | N/A | N/A | N/A | Population maximum allele frequency within non-Finnish Europeans is 0.002105. |
|  | *MAST4* | 5:67100558 A>G | 38 | 0 | Missense | 2 | Het | N/A | N/A | N/A | Not foundon gnomAD population database. |
|  | *PDSS1* | 10:26709669 A>G | 38 | 0 | Missense | 3 | Het | Primary coenzyme Q10 deficiency | 614651 | AR | Best candidate as pigmentary chnages within the retina mentioned in some syndromic primary COQ10 deficiency patients |
|  | *PDSS1* | 10:26697729 G>A | 38 | 0.00001315 | Stop gained | 1 | Het | Primary coenzyme Q10 deficiency | 614651 | AR | Best candidate as pigmentary chnages within the retina mentioned in some syndromic primary COQ10 deficiency patients |
| Family-3  (GC35315) | *PDSS1* | 10:26724012 A>G | 38 | 0 | Splice | 2 | Het | Primary coenzyme Q10 deficiency | 614651 | AR | Best candidate |
|  | *PDSS1* | 10:26720339 A>G | 38 | 0.001793 | Missense | 2 | Het | Primary coenzyme Q10 deficiency | 614651 | AR | Best candidate |
|  | *PNPLA2* | 11:824042 C>T | 38 | 0.0007557 | Missense | 1 | Het | Neutral lipid storage disease with myopathy | 610717 | AR | Unrelated to phenotype |
|  | *PNPLA2* | 11:824794 G>C | 38 | 0.0003879 | Missense | 1 | Het | Neutral lipid storage disease with myopathy | 610718 | AR | Unrelated to phenotype |
|  | *MUC12* | 7:100994318 G>A | 38 | 0 | Missense | 2 | Hom | N/A | N/A | N/A | Excluded; mucin gene; poor coverage region. |
| Family-7  (GC26032) | *COQ2* | 4:83272130 G>C | 38 | 0 | Stop gained | 3 | Het | Primary coenzyme Q10 deficiency Multiple system atrophy | 607426 146500 | AR | Best candidate as pigmentary chnages within the retina mentioned in some syndromic primary COQ10 deficiency patients |
|  | *COQ2* | 4:83273505 T>C | 38 | 0.0000854 | Missense | 8 | Het | Primary coenzyme Q10 deficiency Multiple system atrophy | 607426 146500 | AR | Best candidate as pigmentary chnages within the retina mentioned in some syndromic primary COQ10 deficiency patients |

| Family-10  (GC20389) | *COQ4* | 9:131095818 G>A | 37 | 3.23227E-05 | Missense | N/A | Het | Primary coenzyme Q10 deficiency | 616276 | AR | Best candidate as pigmentary chnages within the retina mentioned in some syndromic primary COQ10 deficiency patients |
| --- | --- | --- | --- | --- | --- | --- | --- | --- | --- | --- | --- |
|  | *COQ4* | 9:131088134 G>A | 37 | 0.000180374 | Missense | N/A | Het | Primary coenzyme Q10 deficiency | 616276 | AR | Best candidate as pigmentary chnages within the retina mentioned in some syndromic primary COQ10 deficiency patients |
|  | *IGHV4-39* | 14:106877816 AACT>A | 37 | 4.28189E-06 | Frameshift | N/A | Hom | N/A | N/A | N/A | Excluded; immunoglobuline gene. |
|  | *KIR3DL3* | 19:55240995 G>A | 37 | 0 | Missense | N/A | Het | N/A | N/A | N/A | Excluded; poor coverage region. |
|  | *KIR3DL3* | 19:55241172 A>C | 37 | 0 | Missense | N/A | Het | N/A | N/A | N/A | Excluded; poor coverage region. |
|  | *MUC17* | 7:100682958 C>T | 37 | 4.33435E-05 | Missense | N/A | Het | N/A | N/A | N/A | Excluded; mucin gene. |
|  | *MUC17* | 7:100684172 C>G | 37 | 6.13718E-05 | Missense | N/A | Het | N/A | N/A | N/A | Excluded; mucin gene. |
|  | *SCML4* | 6:108070978 G>C | 37 | 0.000012183 | Missense | N/A | Hom | N/A | N/A | N/A | Rare biallelic homozygous variant. |
|  | *SRA1* | 5:139931628 A>AGTC | 37 | 0.0000108 | Frameshift | N/A | Het | N/A | N/A | N/A | Excluded; similar frameshifts within the region are common; multiallelic variant. Same genotype is identified in GC21390. *In cis* with 5:139931629 C>G. |
|  | *SRA1* | 5:139931629 C>G | 37 | 0.000263709 | Missense | N/A | Het | N/A | N/A | N/A | Excluded; similar frameshifts within the region are common; multiallelic variant. Same genotype is identified in GC21390. *In cis* with 5:139931628 A>AGTC. |
|  | *TMEM232* | 5:109756292 C>A | 37 | 0.000119909 | Stop gained | N/A | Het | N/A | N/A | N/A | Excluded; *in cis* with 5:109756361 C>G. |
|  | *TMEM232* | 5:109756361 C>G | 37 | 0.000110611 | Missense | N/A | Het | N/A | N/A | N/A | Excluded; *in cis* with 5:109756292 C>A. |
|  | *TTN* | 2:179429764 ATTG>A | 37 | 3.23394E-05 | Frameshift | N/A | Het | Cardiomyopathy Muscular dystrophy Myopathy | 604145 613765 608807 603689 611705 600334 | AD AR AD AR AD | Unrelated to phenotype |
|  | *TTN* | 2:179605911 A>C | 37 | 0.000068876 | Missense | N/A | Het | Cardiomyopathy Muscular dystrophy Myopathy | 604145 613765 608807 603689 611705 600334 | AD AR AD AR AD | Unrelated to phenotype |
|  | *XKR3* | 22:17264565 G>T | 37 | 0 | Missense | N/A | Hom | N/A | N/A | N/A | Excluded; poor coverage region. |
|  | *ZNF813* | 19:53995186 C>T | 37 | 4.06398E-06 | Missense | N/A | Hom | N/A | N/A | N/A | Rare biallelic homozygous variant. |

Supplementary Table 5 (continued)

Supplementary Table 5 (continued)

| Family-11  (GC21390) | *COQ5* | 12:120941637 CG>C | 37 | 0.0000176 | Frameshift | N/A | Het | ?Coenzyme Q10 deficiency, primary | 619028 | AR | Best candidate as pigmentary chnages within the retina mentioned in some syndromic primary COQ10 deficiency patients |
| --- | --- | --- | --- | --- | --- | --- | --- | --- | --- | --- | --- |
|  | *COQ5* | 12:120942793 A>C | 37 | 0.001434 | Splice region | N/A | Het | ?Coenzyme Q10 deficiency, primary | 619028 | AR | Best candidate as pigmentary chnages within the retina mentioned in some syndromic primary COQ10 deficiency patients |
|  | *DNAH1* | 3:52422629 C>T | 37 | 0.0001129 | Splice region | N/A | Het | Ciliary dyskinesia, primary Spermatogenic failiure | 617577 617576 | AR | Unrelated to phenotype |
|  | *DNAH1* | 3:52430472 T>C | 37 | 0.0001929 | Missense | N/A | Het | Ciliary dyskinesia, primary Spermatogenic failiure | 617577 617576 | AR | Unrelated to phenotype |
|  | *SRA1* | 5:139931629 C>G | 37 | 0.000263709 | Missense | N/A | Het | N/A | N/A | N/A | Excluded; similar frameshifts within the region are common; multiallelic variant. Same genotype is identified in GC20389. *In cis* with 5:139931628 A>AGTC. |
|  | *SRA1* | 5:139931628 A>AGTC | 37 | 1.08267E-05 | Frameshift | N/A | Het | N/A | N/A | N/A | Excluded; similar frameshifts within the region are common; multiallelic variant. Same genotype is identified in GC20389. *In cis* with 5:139931629 C>G. |

Supplementary Table 6. PCR primer sequences

| **Gene** | **Name** | **Sequence** |
| --- | --- | --- |
| *PDSS1* | Exon 6 forward | 5'-tcc gat gtc cag ttt tca ca-3' |
|  | Exon 6 reverse | 5'-tga gaa aca aat gcg gtc cg-3' |
|  | Exon 9/10 forward | 5'-cct tca gcc agg ggt cag-3' |
|  | Exon 9/10 reverse | 5'-tgc aat ctt ccc atc agc tg-3' |
| *COQ2* | Exon 1 forward | 5'-ttt cct cag aga ccc cgt tc-3' |
|  | Exon 1 reverse | 5'-ttc tca ttt cca tca cgc cc-3' |
|  | Exon 3 forward | 5'-tac cat ggg cca gtc tct tc-3' |
|  | Exon 3 reverse | 5'-aca ctt gct aac tta cag atg ct-3' |
| *COQ5* | COQ5_missense_variant_F | 5’-aga ggt cat cgc tgg aga ct-3’ |
|  | COQ5_missense_variant_R | 5’-tcc ttc agt tcc agg ctt tca-3’ |

Supplementary Table 7. Plasma CoQ10 status of studies subjects.

| **Family** | **Gene** | **Variants** | **Individual, status** | **CoQ10 (nmol/L)** |
| --- | --- | --- | --- | --- |
| Family-1  (GC3635) | *PDSS1* | c.18G>A, p.(Trp6*) | II:1, Affected | 256.34 |
|  |  | c.368A>G, p.(Glu123Gly) |  |  |
|  | *PDSS1* | c.18G>A, p.(Trp6*) | II:2, Heterozygote | 394.9 |
| Family-2  (GC25311) | *PDSS1* | c.232C>A, p.(His78Asn) | II:1, Affected | 461.81 |
|  |  | c.886G>A, p.(Gly296Arg) |  |  |
| Family-3  (GC35315) | *PDSS1* | c.589A>G, p.(Lys197Glu) | II:2, Affected | 258.93 |
|  |  | c.722-2A>G, p.(Ala204_Ala277del); p.(Gly241Alafs*6) |  |  |
|  | *PDSS1* | c.722-2A>G, p.(Ala204_Ala277del); p.(Gly241Alafs*6) | I:2, Heterozygote | 454.1 |
|  | *PDSS1* | c.589A>G, p.(Lys197Glu) | I:1, Heterozygote | 347.57 |
| Family-5 | *PDSS1* | c.468-25A>G, p.? | II:1, Affected | 750.9 |
|  |  | c.589A>G, p.(Lys197Glu) |  |  |
| Family-6  (ARRP195) | *PDSS1* | c.589A>G, p.(Lys197Glu) | II:1, Affected | 195.45 |
|  |  | c.893dup, p.(Asn298Lysfs*11) |  |  |
| Family-7  (GC26032) | *COQ2* | c.683A>G, p.(Asn228Ser) | II:1, Affected | 39.33 |
|  |  | c.735C>G, p.(Tyr245*) |  |  |
| Family-8  (SRP1143) | *COQ2* | c.338_341delinsG, p.(Val113_114delinsGly) | II:1, Affected | 410.23 |
|  |  | c.683A>G, p.(Asn228Ser) |  |  |
| Family-10  (GC20389) | *COQ4* | c.376G>A, p.(Glu126Lys ) | II:5, Affected | 582.28 |
|  |  | c.692G>A, p.(Cys231Tyr) |  |  |
| Family-11  (GC21390) | *COQ5* | c.682-7T>G, p.(Gln230*); p.(Leu193Phefs*27) | II:1, Affected | 582.28 |
|  |  | c.933delC, p.(Tyr311*) |  |  |

Reference range: 227- 1432 nmol/L.

*
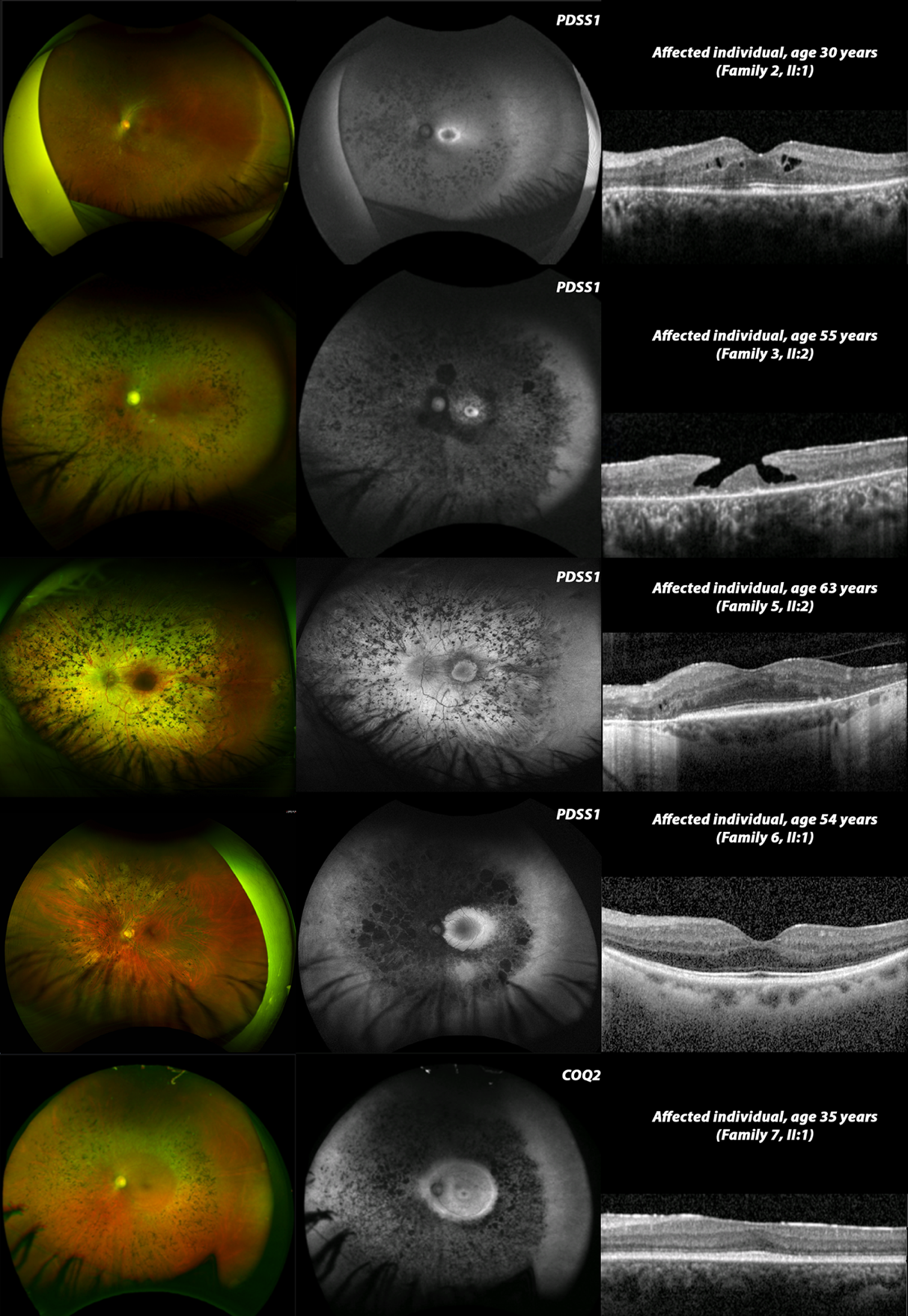
*

Supplementary Figure 1. Multimodal imaging of affected individuals (left eye only) carrying candidate pathogenic variants in *PDSS1* and *COQ2*. Ultra-widefield fundus pseudocolor imaging shows characteristic features of retinitis pigmentosa: retinal vessels attenuation, pigmentary (bone spicule) changes in mid-periphery. Ultra-widefield fundus autofluorescence image demonstrates hypoautofluorescence corresponding to mid-peripheral bone spicules with a hyperautofluorescence ring at the macula delineating the border between normal (inside the ring) and abnormal retina (outside the ring). SD-OCT macula scan through the fovea shows absence of inner segment/outer segment (IS/OS) junction in the parafoveal region in affected individuals carrying candidate pathogenic variants in *PDSS1* and *COQ2*. Cystoid macular oedema (Family 2, II:2), decreased choroidal thickness (Family 5, II:1 and family 7, II:1), mild epiretinal membrane (Family 7, II:1) and lamellar hole subsequent upon to post epiretinal membrane peeling (Family 3, II:2) depicted in the affected individuals.

*
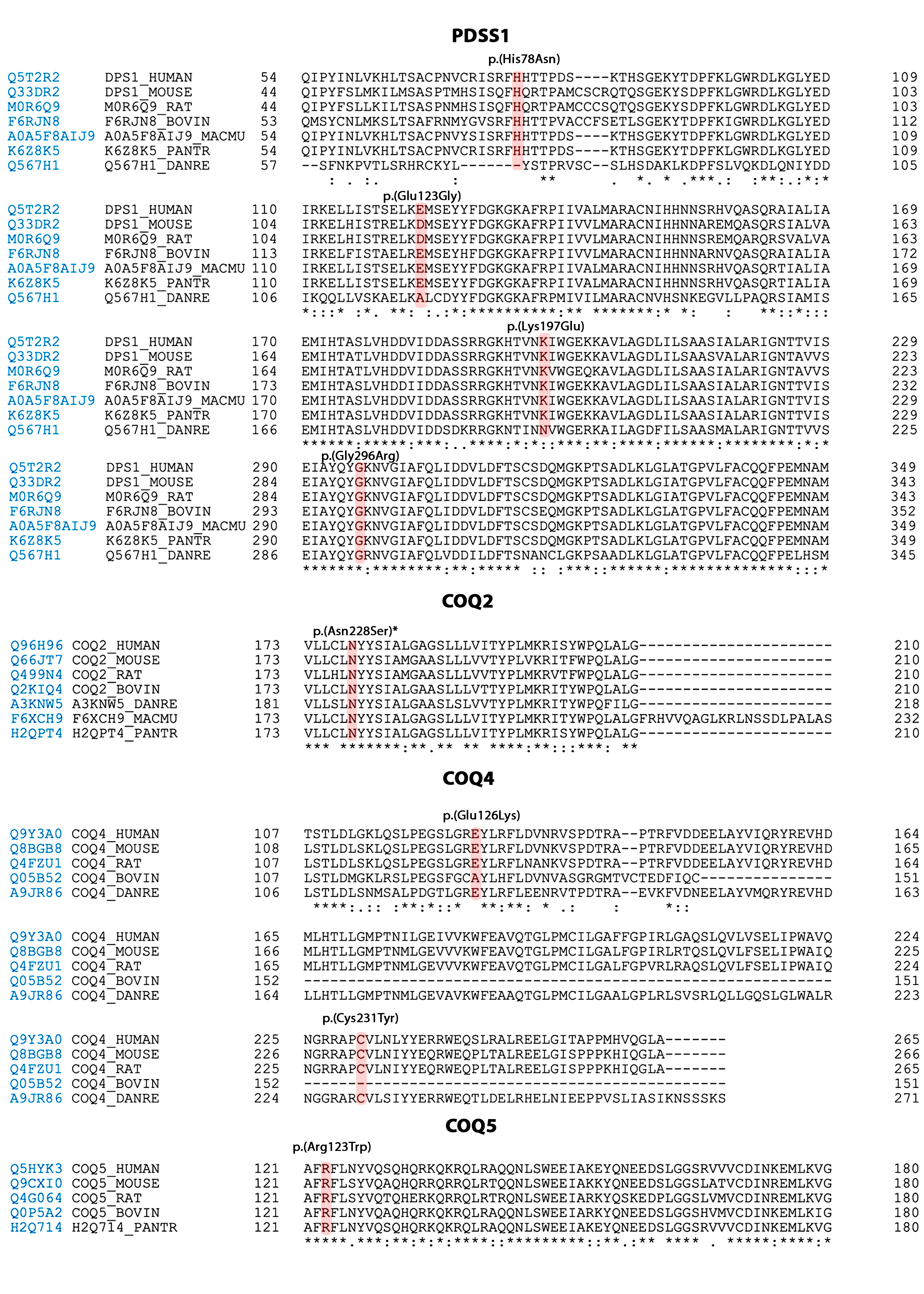
*

Supplementary Figure 2. Multiple alignment of PDSS1, COQ2, COQ4 and COQ5 orthologues. Residues affected by missense variants are highlighted. *Variant position for NM_015697.9
